# Associations between plasma kynurenines and cognitive function in individuals with normal glucose metabolism, prediabetes and type 2 diabetes: the Maastricht Study

**DOI:** 10.1007/s00125-021-05521-4

**Published:** 2021-08-19

**Authors:** Lieke Bakker, Inez H. G. B. Ramakers, Martin P. J. van Boxtel, Miranda T. Schram, Coen D. A. Stehouwer, Carla J. H. van der Kallen, Pieter C. Dagnelie, Marleen M. J. van Greevenbroek, Anke Wesselius, Øivind Midttun, Per M. Ueland, Frans R. J. Verhey, Simone J. P. M. Eussen, Sebastian Köhler

**Affiliations:** 1 MHeNs School for Mental Health and Neuroscience, Maastricht University, Maastricht, the Netherlands; 2 Department of Psychiatry and Neuropsychology, Alzheimer Center Limburg, Maastricht University, Maastricht, the Netherlands; 3 CARIM School for Cardiovascular Diseases, Maastricht University, Maastricht, the Netherlands; 4 Department of Internal Medicine, Maastricht University Medical Center+, Maastricht, the Netherlands; 5 Heart and Vascular Center, Maastricht University Medical Center+, Maastricht, the Netherlands; 6 CAPHRI School for Public Health and Primary Care, Maastricht University, Maastricht, the Netherlands; 7 Department of Complex Genetics and Epidemiology, Maastricht University, Maastricht, the Netherlands; 8 Bevital AS, Bergen, Norway; 9 University of Bergen, Bergen, Norway; 10 Haukeland University Hospital, Bergen, Norway; 11 Department of Epidemiology, Maastricht University, Maastricht, the Netherlands

**Keywords:** Cognition, Cognitive impairment, Glucose metabolism status, Kynurenines, Metabolites, Population-based cohort study, Prediabetes, Type 2 diabetes mellitus

## Abstract

**Aims/hypothesis:**

Studies investigating associations between kynurenines and cognitive function have generally been small, restricted to clinical samples or have found inconsistent results, and associations in the general adult population, and in individuals with type 2 diabetes in particular, are not clear. Therefore, the aim of the present study was to investigate cross-sectional associations between plasma kynurenines and cognitive function in a cohort of middle-aged participants with normal glucose metabolism, prediabetes (defined as impaired fasting glucose and/or impaired glucose tolerance) and type 2 diabetes.

**Methods:**

Plasma kynurenines were quantified in 2358 participants aged 61 ± 8 years. Cross-sectional associations of kynurenines with cognitive impairment and cognitive domain scores were investigated using logistic, multiple linear and restricted cubic spline regression analyses adjusted for several confounders.

**Results:**

Effect modification by glucose metabolism status was found for several associations with cognitive impairment, hence analyses were stratified. In individuals with prediabetes, 3-hydroxykynurenine (OR per SD 0.59 [95% CI 0.37, 0.94]) and 3-hydroxyanthranilic acid (0.67 [0.47, 0.96]) were associated with lower odds of cognitive impairment after full adjustment. In individuals with type 2 diabetes, kynurenine (0.80 [0.66, 0.98]), 3-hydroxykynurenine (0.82 [0.68, 0.99]), kynurenic acid (0.81 [0.68, 0.96]), xanthurenic acid (0.73 [0.61, 0.87]) and 3-hydroxyanthranilic acid (0.73 [0.60, 0.87]) were all associated with lower odds of cognitive impairment. Kynurenic acid (β per SD 0.07 [95% CI 0.02, 0.13]) and xanthurenic acid (0.06 [0.01, 0.11]) were also associated with better executive function/attention. No associations were observed in individuals with normal glucose metabolism.

**Conclusions/interpretation:**

Several kynurenines were cross-sectionally associated with lower odds of cognitive impairment and better cognitive functioning in type 2 diabetes, while less widespread associations were seen in prediabetes. Low levels of kynurenines might be involved in the pathway of type 2 diabetes and cognitive decline but this needs further studies.

**Graphical abstract:**

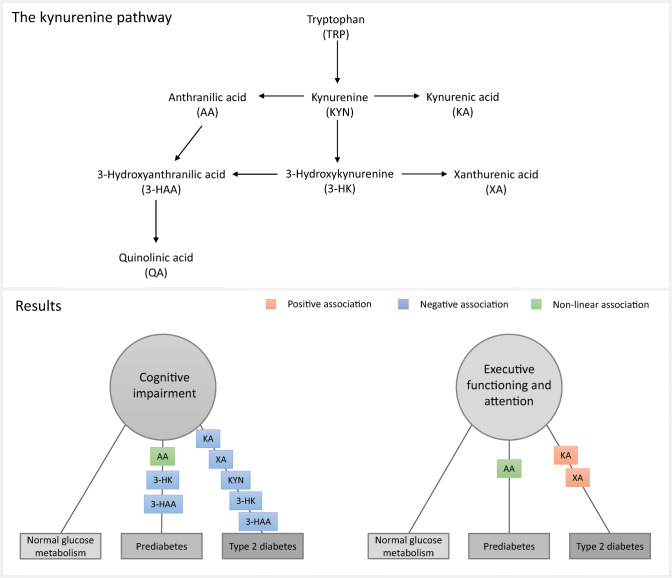

**Supplementary Information:**

The online version of this article (10.1007/s00125-021-05521-4) contains peer-reviewed but unedited supplementary material.

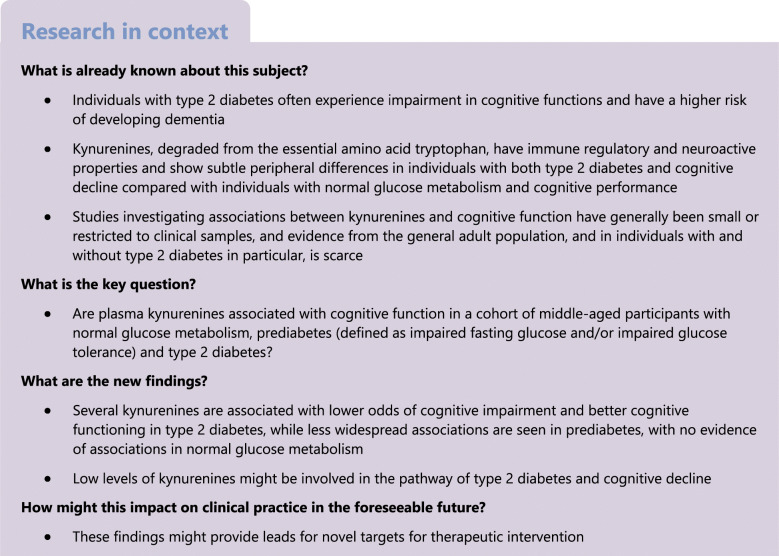



## Introduction

Individuals with type 2 diabetes often show impairment in cognitive functions [1] and have a 60% higher risk of developing dementia [2]. This risk is increased for both Alzheimer’s disease and vascular dementia, the two most common causes of dementia, but appears higher for the latter [3]. Consistently, individuals with type 2 diabetes show early pathophysiological changes associated with cognitive decline and dementia, including microvascular changes and neurodegeneration [4, 5]. These changes can be found as early as the prediabetic stage [6, 7]. The underlying biological mechanisms are not well understood but might include chronic inflammatory processes, oxidative stress and ischaemic damage due to hypertension, atherosclerosis and endothelial dysfunction [8, 9].

**Fig. 1:**
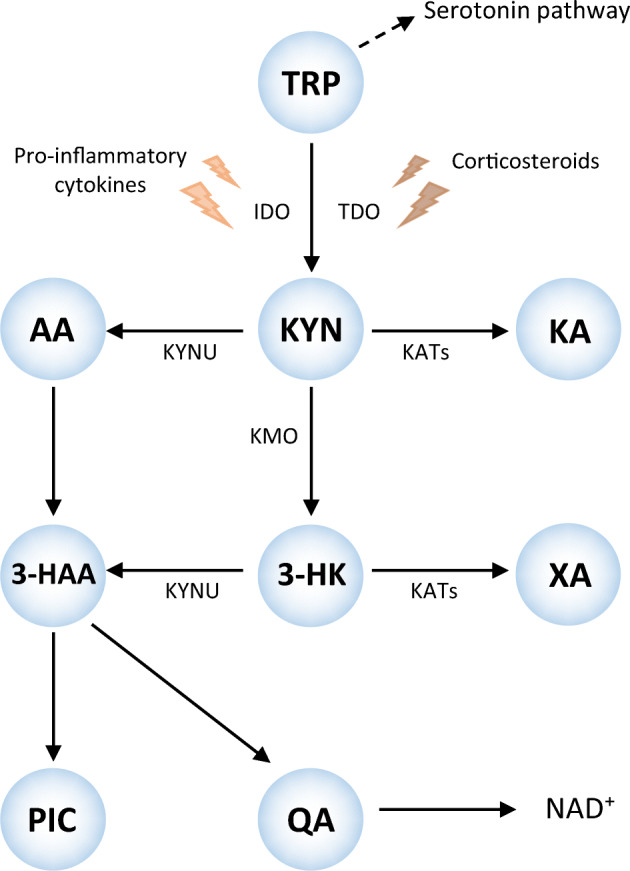
The kynurenine pathway. TRP is converted to KYN by the enzymes IDO and TDO, as reflected by KTR. The KTR increases in response to inflammation, particularly through IFN-γ, which also increases neopterin, a pteridine. KYN can be further degraded into KA, 3-HK and AA by, respectively, the enzymes kynurenine aminotransferase (KAT), kynurenine 3-monooxygenase (KMO) and kynureninase (KYNU); 3-HAA and XA can be synthesised from 3-HK, QA and picolinic acid (PIC) from 3-HAA and, finally, QA is synthesised into nicotinamide adenine dinucleotide (NAD^+^). Riboflavin and PLP, active forms of, respectively, vitamin B_2_ and vitamin B_6_ are cofactors of enzymes of the kynurenine pathway

The tryptophan (TRP)–kynurenine (KYN) pathway (Fig. 1) is involved in all of these processes and might thus play a role in the association between type 2 diabetes and cognition, as subtle differences in peripheral concentrations have been observed in individuals with insulin resistance or type 2 diabetes compared with healthy individuals [10, 11]. TRP is an essential amino acid that has an important role in brain-related functions, including protein formation, and as a precursor for the serotonergic and KYN pathway, the latter being the main metabolic route [12]. Following hepatic tryptophan 2,3-dioxygenase (TDO) activation, TRP is largely converted to the central metabolite KYN, and into further downstream metabolites, collectively known as kynurenines, which have immune regulatory and neuroactive properties [12]. Kynurenic acid (KA) is known to be neuroprotective due to its anti-inflammatory and anti-glutamatergic effects. Other kynurenines, including quinolinic acid (QA), have neurotoxic properties through their role in glutamate excitotoxicity, neuroinflammation and oxidative stress among other processes [12]. Previous studies that have focused on clinical samples of patients with cognitive impairment and dementia have often reported an imbalance in neurotoxic-to-neuroprotective metabolites [13–17], and one prospective study has found an association between elevated levels of anthranilic acid (AA) and risk for dementia and Alzheimer’s disease dementia [18]. However, in a recent population-based study in healthy older adults, no associations between concentrations of kynurenines and cognitive test scores were found [19].

As these studies generally have been small [13, 15–17], been restricted to clinical samples [13–17] or shown inconsistent results, the relationship between metabolites of the KYN pathway and cognitive function in the general adult population, and particularly in individuals with type 2 diabetes, is unclear. The present study therefore aims to elucidate the associations between a comprehensive panel of plasma kynurenines and cognitive function in a cohort of middle-aged individuals. Since both dysregulations of the KYN metabolic pathway and a higher risk of dementia have been found in type 2 diabetes [2, 10], we also investigate whether these associations are modified by glucose metabolism status.

## Methods

### The Maastricht Study: population and design

We used data from the Maastricht Study, an observational, prospective, population-based cohort study. The rationale and methodology have been described previously [20]. In brief, this study focuses on the aetiology, pathophysiology, complications and comorbidities of type 2 diabetes and is characterised by an extensive phenotyping approach. Eligible for participation were individuals between 40 and 75 years of age living in the southern part of the Netherlands. Participants were recruited through mass media campaigns and from the municipal registries and the regional Diabetes Patient Registry via mailings. Recruitment was stratified according to known type 2 diabetes status, with an oversampling of individuals with type 2 diabetes for reasons of efficiency. The present report includes cross-sectional data from 2358 participants who completed the baseline survey between November 2010 and September 2013 (flowchart is shown in Electronic supplementary material [ESM] Fig. 1; 2358 participants remained in the cohort after excluding participants with no kynurenine concentrations [*n* = 999] and those with no cognitive data available [*n* = 94]). The examination of each participant was performed within a time window of 3 months. The study was approved by the institutional medical ethics committee (NL31329.068.10) and the Minister of Health, Welfare and Sport of the Netherlands (permit 131088–105234-PG) and was conducted in accordance with the Declarations of Helsinki. All participants gave written informed consent.

### Glucose metabolism status

To determine glucose metabolism status, all participants (except those who used insulin) underwent an OGTT after an overnight fast as previously described [20]. A distinction into four different categories was used according to the WHO 2006 criteria [21]: 1, normal glucose metabolism (NGM); 2, impaired fasting glucose; 3, impaired glucose tolerance; and 4, type 2 diabetes. Categories 2 and 3 were considered as ‘prediabetes’.

### Blood samples and biochemical analysis

Kynurenines, neopterin and vitamins B_2_ and B_6_ in plasma were determined in a single aliquot from 2452 participants, including all participants with prediabetes (*n* = 509) and type 2 diabetes (*n* = 956) and a random sample from those with NGM (*n* = 986). Fasting blood samples were collected in EDTA tubes. After centrifuging, plasma was stored at −80°C.

Concentrations of TRP (μmol/l) and seven kynurenines (KYN [μmol/l], 3-hydroxykynurenine [3-HK, nmol/l], KA [nmol/l], xanthurenic acid [XA, nmol/l], anthranilic acid [AA, nmol/l], 3-hydroxyanthranilic acid [3-HAA, nmol/l] and QA [nmol/l]) were determined in 200 μl plasma samples by LC-MS/MS (Bevital, Bergen Norway) (http://www.bevital.no). From these concentrations, the KYN/TRP ratio (KTR = [KYN/TRP] × 1000) and KA/QA ratio were calculated. KTR is a measure of cytokine-induced indoleamine 2,3-dioxygenase activity, whereas KA/QA is a potential neuroprotective index [16]. Within-day CVs for the KYN metabolites and B vitamins were 1.8–9.5%, between-day CVs were 4.9–16.9%, and limits of detection were 0.2–7 nmol/l [22].

### Cognitive performance

Cognitive performance was assessed in a concise (30 min) neuropsychological test battery [20]. For conceptual clarity, test scores were standardised and divided into three cognitive domains (memory function, information processing speed, and executive function/attention). Briefly, memory function was evaluated using the Verbal Learning Test [23] and a memory domain score was derived by calculating the average of total immediate and delayed recall standardised scores. An information processing speed domain score was derived from standardised scores of the Stroop Colour-Word Test Part I and II [24], the Concept Shifting Test Part A and B [25] and the Letter-Digit Substitution Test [26]. An executive function/attention domain score was derived from the Stroop Colour-Word Test part III (adjusted for the mean time spend on the first two parts, Stroop I and II) and Concept Shifting Test Part C. Each of the cognitive domains was only calculated if all subtests were completed. If necessary, individual test scores were log-transformed to reduce skewness of distributions and/or inverted so that higher scores indicated better cognitive performance. In addition, participants were categorised as cognitively impaired (yes/no) based on a regression-based normalisation procedure per test that predicted expected scores for each individual given their age, sex and level of education from a published normative sample [23–26]. The difference between observed and expected scores and their SDs were used to calculate *z* scores, which were then averaged per domain and re-standardised. Individuals performing < −1.5 SDs below their norm-based expected score in any domain were categorised as having significant cognitive impairment (CogImp).

### General characteristics and covariates

As previously described [20], educational level (low, intermediate, high), smoking status (never, current, former), alcohol consumption (none, low, high), physical activity and history of CVD were assessed by questionnaires. We measured BMI, office BP, serum creatinine and cystatin C as described elsewhere [20]. eGFR (in ml min^−1^ 1.73 m^−2^) was calculated with the Chronic Kidney Disease Epidemiology Collaboration equation based on both serum creatinine and serum cystatin C [27]. The Mini International Neuropsychiatric Interview [28] was used to identify the presence of a major depressive disorder (yes/no). Medication use was assessed in an interview where generic name, dose and frequency were registered. Serum HDL-cholesterol (mmol/l) and total cholesterol were also determined. C-reactive protein (CRP, μg/ml), serum amyloid A (SAA, μg/ml), soluble intercellular adhesion molecule-1 (sICAM-1, ng/ml), IL-6 (pg/ml), IL-8 (pg/ml) and TNF-α (pg/ml) were determined in EDTA plasma by 4-plex sandwich immunoassays from MSD (USA). CRP was quantified on a 4-plex with SAA and sICAM-1, whereas IL-6 was quantified on a 4-plex together with IL-8 and TNF-α.

### Statistical analyses

One-way ANOVA and χ^2^ tests were used to investigate differences in characteristics between participants with and without cognitive impairment and between participants with NGM status, prediabetes and type 2 diabetes. Next, logistic and multiple linear regression models were used to test the associations of plasma kynurenines (as main independent variables) with cognitive impairment and cognitive domain scores (as main dependent variables). For reasons of comparison, metabolite levels were standardised prior to analysis. To investigate potential non-linear associations, we used restricted cubic spline regression models with four knots (fixed knots at *z* scores of −2, 0, 2, 4) [29]. This method avoids arbitrary categorisation of the continuous measures of kynurenines [30]. Goodness of fit of these models was determined by likelihood ratio tests and visual inspection of smoothed spline plots. Analyses were exploratory and not controlled for multiple testing.

The effect of covariates adjustment on the association was tested in the following models: (1) demographics (age, sex, educational level) and eGFR; and (2) model 1 + lifestyle factors (BMI, cholesterol ratio [total cholesterol/HDL-cholesterol], lipid-modifying medication use, alcohol consumption and smoking behaviour). We expected different associations in participants with NGM, prediabetes and type 2 diabetes, and studies suggest that kynurenine concentrations increase with age [14, 16]. Therefore, interaction analyses investigated whether associations between concentrations of kynurenines and cognitive function were modified by glucose metabolism status, age or sex. Unless otherwise stated, results are presented for main model 2.

B vitamins, inflammatory markers and cardiovascular risk factors are potential mediators along the pathway of kynurenine concentrations and cognitive function [31]. Therefore, additional adjustment for vitamin B_2_ and B_6_ species riboflavin and pyridoxal 5′-phosphate (PLP), low-grade inflammation (composite score of CRP, SAA, sICAM-1, IL-6, IL-8 and TNF-α; transferred into *z* scores and averaged), hypertension, history of CVD, current depressive episode and antidepressant use (model 3) was done in sensitivity analyses to avoid overcorrection bias. To further test the robustness of associations, additional sensitivity analyses were done: one model in which current depression was replaced by lifetime depression; one model in which antidepressant use was removed as this medication is also used for indications other than depression; one model in which hypertension was replaced by systolic BP and hypertension medication; and one model in which physical activity was added to model 3. For all analyses, IBM SPSS 22.0 (IBM, USA) and STATA 13 (StataCorp, USA) were used with a two-sided *p* level of <0.05 considered statistically significant.

## Results

### Characteristics of study population

Within the sample with measured kynurenine concentrations, data on cognitive function was available for 2358 participants (96.2% of full sample, 54.5% men, mean age 60.6 ± 8.0 years). Of this group, 440 (18.7%) participants were classified as cognitively impaired. Compared with the group without cognitive impairment, they were more often men, had a lower eGFR, had a higher BMI, were less physically active, were more often current smokers and consumed less alcohol (Table 1). They also had a higher prevalence of type 2 diabetes, hypertension, a history of CVD and depression, and used lipid-modifying agents, antihypertensive agents and antidepressants more often. Lastly, they had lower plasma concentrations of TRP, XA and PLP, higher KTR, higher plasma concentrations of neopterin and sICAM, and greater presence of low-grade inflammation (Table 2).

**Table 1:** General characteristics and cognitive domain scores of study participants with and without cognitive impairment

Characteristic	Total (*n*=2358)	Cognitive intact (*n*=1854)	Cognitive impairment (*n*=440)	*p* value
Demographics				
Age, years	60.6 ± 8.0	60.4 ± 8.0	61.2 ± 7.9	0.061
Men, *n* (%)	1285 (54.5)	978 (52.8)	271 (61.6)	0.001
Educational level, *n* (%)				0.184
Low	833 (35.3)	667 (36.0)	159 (36.1)	
Intermediate	661 (28.0)	518 (27.9)	140 (31.8)	
High	810 (34.4)	666 (35.9)	141 (32.0)	
Lifestyle factors				
Smoking, *n* (%)				<0.001
Never	768 (32.6)	613 (33.1)	146 (33.2)	
Former	1241 (52.6)	1019 (55.0)	209 (47.5)	
Current	304 (12.9)	213 (11.5)	82 (18.6)	
Alcohol use, *n* (%)				<0.001
None	466 (19.8)	339 (18.3)	119 (27.0)	
Low	1254 (53.2)	1015 (54.7)	227 (51.6)	
High	589 (25.0)	488 (26.3)	90 (20.5)	
Physical activity (h/week)	13.7 ± 8.0	14.0 ± 7.9	12.7 ± 8.5	0.005
Cardiovascular risk factors				
BMI, kg/m^2^	27.6 ± 4.7	27.5 ± 4.7	28.3 ± 4.8	0.002
Systolic BP, mmHg	136.6 ± 18.2	136.2 ± 18.0	138.0 ± 18.7	0.055
Diastolic BP, mmHg	76.5 ± 9.8	76.5 ± 9.6	76.5 ± 10.3	0.977
eGFR, ml min^−1^ 1.73 m^−2^	87.5 ± 15.2	88.1 ± 14.6	85.3 ± 17.4	0.001
Total/HDL-cholesterol ratio	3.72 ± 1.20	3.73 ± 1.21	3.74 ± 1.18	0.782
Diseases, *n* (%)				
Prediabetes	495 (21.0)	404 (21.7)	78 (17.7)	0.063
Type 2 diabetes	905 (38.4)	654 (35.3)	214 (48.6)	<0.001
Hypertension	1449 (61.5)	1109 (59.8)	302 (68.6)	<0.001
History of CVD	415 (17.6)	295 (15.9)	113 (25.7)	<0.001
Depression	102 (4.3)	60 (3.2)	40 (9.1)	<0.001
Medication use, *n* (%)				
Lipid-modifying agent	1001 (42.5)	738 (39.8)	232 (52.7)	<0.001
Antihypertensive agent	1077 (45.7)	810 (43.7)	239 (54.3)	<0.001
Antidepressant	173 (7.3)	122 (6.6)	46 (10.5)	0.005
Cognitive domain score				
Memory	−0.07 ± 0.97	0.17 ± 0.85	−0.88 ± 0.98	<0.001
Information processing	−0.05 ± 0.79	0.09 ± 0.66	−0.79 ± 0.80	<0.001
Executive function/attention	−0.06 ± 0.81	0.10 ± 0.69	−0.73 ± 0.94	<0.001

Data are presented as *n* (%) or mean ± SD

For 64 participants, information on the presence of cognitive impairment was not available

One-way ANOVA and χ^2^ tests were used to investigate differences in characteristics between participants with and without cognitive impairment

**Table 2:** Concentrations of kynurenines, inflammation markers and B vitamins of study participants with and without cognitive impairment

Characteristic	Total (*n*=2358)	Cognitive intact (*n*=1854)	Cognitive impairment (*n*=440)	*p* value
Metabolite levels				
TRP, *μ*mol/l	62.80 (56.60–69.50)	62.90 (56.80–69.80)	61.95 (55.80–68.68)	0.033
KYN, *μ*mol/l	1.64 (1.41–1.92)	1.63 (1.40–1.92)	1.66 (1.45–1.94)	0.463
3-HK, nmol/l	41.90 (34.50–50.40)	41.80 (34.70–50.10)	42.30 (34.20–53.23)	0.211
KA, nmol/l	52.50 (41.40–66.23)	52.30 (41.40–65.80)	53.75 (41.15–67.70)	0.235
XA, nmol/l	14.30 (10.50–18.70)	14.50 (10.68–18.80)	13.20 (9.59–18.28)	0.031
AA, nmol/l	14.80 (12.30–18.00)	14.80 (12.30–18.00)	14.80 (12.08–18.10)	0.825
3-HAA, nmol/l	37.70 (29.80–47.60)	38.10 (30.20–47.70)	36.90 (28.25–46.60)	0.101
QA, nmol/l	388.0 (314.0–492.3)	388.0 (314.0–489.3)	393.0 (313.3–506.0)	0.913
Ratios				
KTR	25.87 (22.27–30.49)	25.56 (22.18–30.27)	26.40 (22.61–31.56)	0.031
KA/QA	0.133 (0.106–0.167)	0.133 (0.106–0.166)	0.132 (0.107–0.169)	0.524
Low-grade inflammation^a^	0.05 ± 0.66	0.02 ± 0.65	0.18 ± 0.67	<0.001
Inflammation markers				
Neopterin, nmol/l	16.61 (13.77–20.29)	16.41 (13.65–19.98)	17.12 (14.30–21.59)	0.002
CRP, μg/ml	1.33 (0.63–3.00)	1.31 (0.63–2.99)	1.43 (0.66–3.12)	0.447
SAA, μg/ml	3.33 (2.08–5.54)	3.32 (2.08–5.43)	3.43 (2.09–6.16)	0.800
sICAM-1, ng/ml	342.6 (294.5–404.0)	339.6 (291.4–399.8)	357.7 (303.0–417.2)	<0.001
IL-6, pg/ml	0.63 (0.42–0.96)	0.61 (0.41–0.93)	0.72 (0.49–1.14)	0.157
IL-8, pg/ml	4.27 (3.42–5.51)	4.22 (3.38–5.44)	4.43 (3.55–5.88)	0.727
TNF-α, pg/ml	2.22 (1.91–2.61)	2.21 (1.89–2.57)	2.33 (1.97–2.82)	0.210
B vitamins, nmol/l				
PLP	57.90 (40.50–85.83)	58.35 (41.50–86.50)	54.95 (36.88–84.13)	0.030
Riboflavin	13.50 (8.75–22.10)	13.60 (8.90–22.40)	12.50 (8.28–21.20)	0.186

Data are presented as median (IQR) or mean ± SD

For 64 participants, information on the presence of cognitive impairment was not available

^a^Composite score of CRP, SAA, sICAM-1, IL-6, IL-8 and TNF-α (transferred into *z* scores and averaged)

One-way ANOVA was used to investigate differences in characteristics between participants with and without cognitive impairment

General characteristics and metabolite levels by glucose metabolism status are provided in ESM Tables 1 and 2. When considering differences between participants according to glucose metabolism status, those with prediabetes and type 2 diabetes were significantly older, more often men, had a lower educational level, a lower eGFR, a lower total cholesterol/HDL-cholesterol ratio, a higher BMI, higher systolic and diastolic BP, were more often former or current smokers and scored lower on all three cognitive domain scores compared with the group with NGM. They also had a higher prevalence of hypertension, more often used lipid-modifying agents, antihypertensive agents and antidepressants and had lower plasma concentrations of PLP together with higher plasma concentrations of most kynurenines, inflammation markers and KTR. Lastly, the group with type 2 diabetes also used less alcohol, were less physically active, had a higher prevalence of depression, a history of CVD and cognitive impairment and higher plasma concentrations of IL-6 and TNF-α compared with those with NGM (ESM Tables 1 and 2).

### Effect modification by glucose metabolism status

Interaction analyses tested whether associations between plasma kynurenines and cognition differed between participants with NGM, prediabetes and type 2 diabetes in main model 2 (ESM Table 3). These showed significant and directionally consistent effect modification by glucose metabolism status for the associations between XA (*p*_interaction_ = 0.027), AA (*p*_interaction_ = 0.048) and 3-HAA (*p*_interaction_ = 0.030) and odds of cognitive impairment. We therefore decided to present stratified analyses for all kynurenines.

**Fig. 2:**
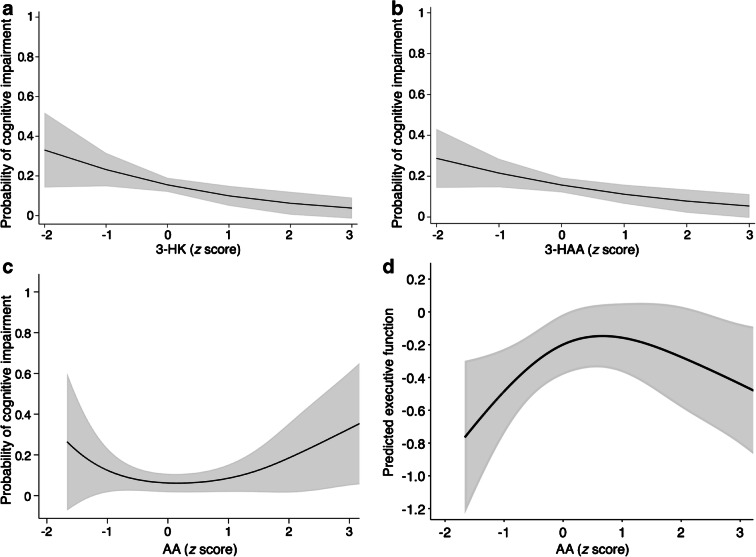
Associations of kynurenines with cognitive impairment (**a**–**c**) and executive function/attention domain scores (**d**) in participants with prediabetes. Metabolite concentrations were standardised prior to analysis and all analyses were adjusted for age, sex, educational level, eGFR, BMI, total cholesterol/HDL-cholesterol ratio, lipid-modifying medication use, alcohol consumption and smoking behaviour. Shaded areas represent the 95% CI

**Fig. 3:**
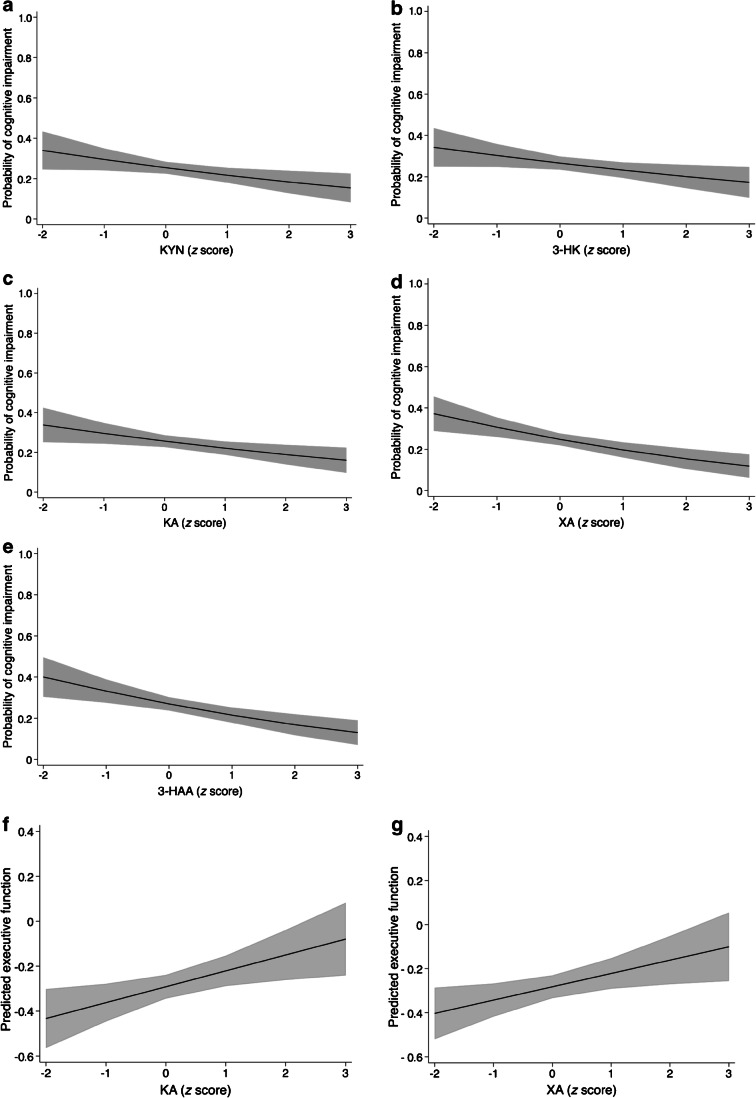
Associations of kynurenines with cognitive impairment (**a**–**e**) and executive function/attention domain scores (**f**, **g**) in participants with type 2 diabetes. Metabolite concentrations were standardised prior to analysis and all analyses were adjusted for age, sex, educational level, eGFR, BMI, total cholesterol/HDL-cholesterol ratio, lipid-modifying medication use, alcohol consumption and smoking behaviour. Shaded areas represent the 95% CI

In participants with prediabetes, higher concentrations of 3-HK and 3-HAA were associated with lower odds for cognitive impairment in model 2 (Fig. 2 and Table 3). Additionally, in prediabetes, a non-linear association was found with AA, suggesting that both higher and lower levels of AA were associated with higher odds of cognitive impairment and lower scores on the executive function/attention domain. In type 2 diabetes, higher concentrations of KYN, 3-HK, KA, XA and 3-HAA were all significantly associated with lower odds of cognitive impairment (Fig. 3 and Table 3). Looking at domain-specific associations, those with both KA and XA seemed to be driven by executive function/attention (β per SD [95% CI] 0.07 [0.02, 0.13] and 0.06 [0.01, 0.11], respectively) (ESM Table 4). In the group with NGM, no associations with cognitive impairment were found. There were also no significant interactions of concentrations of kynurenines with age or sex in model 2 (ESM Table 3; *p*_interaction_ < 0.05).

**Table 3:** Association between TRP, kynurenines (and ratios) and cognitive impairment, after controlling for covariates

Variable	NGM	Prediabetes	Type 2 diabetes
TRP			
Model 1^a^	1.03 (0.84, 1.25)	0.82 (0.62, 1.08)	0.88 (0.75, 1.02)
Model 2^b^	1.00 (0.82, 1.23)	0.80 (0.60, 1.07)	0.91 (0.78, 1.06)
Metabolites			
KYN			
Model 1^a^	1.11 (0.88, 1.40)	0.90 (0.67, 1.20)	0.76 (0.63, 0.92)**
Model 2^b^	1.07 (0.84, 1.36)	0.92 (0.67, 1.25)	0.80 (0.66, 0.98)*
3-HK			
Model 1^a^	1.33 (1.02, 1.73)*	0.55 (0.35, 0.87)*	0.82 (0.69, 0.98)*
Model 2^b^	1.22 (0.92, 1.61)	0.59 (0.37, 0.94)*	0.82 (0.68, 0.99)*
KA			
Model 1^a^	0.97 (0.74, 1.26)	1.11 (0.81, 1.52)	0.76 (0.64, 0.91)**
Model 2^b^	0.94 (0.71, 1.24)	1.22 (0.87, 1.70)	0.81 (0.68, 0.96)*
XA			
Model 1^a^	1.04 (0.85, 1.27)	0.75 (0.56, 1.01)	0.68 (0.57, 0.81)***
Model 2^b^	1.02 (0.83, 1.26)	0.78 (0.58, 1.06)	0.73 (0.61, 0.87)***
AA			
Model 1^a^	0.80 (0.59, 1.06)	^c^	0.85 (0.70, 1.02)
Model 2^b^	0.81 (0.60, 1.08)	^c^	0.90 (0.75, 1.09)
3-HAA			
Model 1^a^	1.04 (0.82, 1.32)	0.65 (0.47, 0.91)*	0.70 (0.59, 0.84)***
Model 2^b^	0.99 (0.76, 1.29)	0.67 (0.47, 0.96)*	0.73 (0.60, 0.87)**
QA			
Model 1^a^	0.95 (0.76, 1.19)	0.73 (0.49, 1.08)	0.80 (0.65, 0.98)*
Model 2^b^	0.93 (0.74, 1.19)	0.74 (0.49, 1.11)	0.84 (0.67, 1.05)
Ratios			
KTR			
Model 1^a^	1.13 (0.88, 1.45)	1.00 (0.75, 1.35)	0.88 (0.74, 1.04)
Model 2^b^	1.11 (0.85, 1.45)	1.05 (0.77, 1.42)	0.90 (0.75, 1.08)
KA/QA			
Model 1^a^	1.01 (0.98, 1.04)	0.99 (0.96, 1.03)	0.99 (0.96, 1.01)
Model 2^b^	0.99 (0.81, 1.20)	1.26 (0.99, 1.59)	0.93 (0.79, 1.08)
Neopterin			
Model 1^a^	0.99 (0.77, 1.29)	1.06 (0.79, 1.44)	1.04 (0.89, 1.22)
Model 2^b^	0.98 (0.75, 1.29)	1.09 (0.80, 1.48)	1.04 (0.89, 1.23)

Data are presented as OR per SD (95% CI)

One of the participants had a diagnosis of type 1 diabetes and was excluded when the data was stratified according to glucose metabolism status

^a^Model 1: adjusted for age, sex, educational level and eGFR (*n*=2296)

^b^Model 2: model 1 + BMI, cholesterol ratio, lipid-modifying medication use, alcohol consumption and smoking behaviour (*n*=2274)

^c^Associations were non-linear according to likelihood ratio test and visual inspection

*p<0.05, **p<0.01 and ***p<0.001

### Additional analyses

ssociations were essentially not modified by further adjustment for current depressive episode, antidepressant use, history of CVD, hypertension, B vitamins (PLP, riboflavin) and low-grade inflammation (composite score of CRP, SAA, sICAM-1, IL-6, IL-8 and TNF-α) (ESM Tables 4 and 5). In prediabetes, an association was found between higher KA/QA ratio and higher odds of cognitive impairment. In type 2 diabetes, the initially inverse association between 3-HK and odds for cognitive impairment became non-significant.

Results also remained virtually unchanged by replacing hypertension with systolic BP and hypertension medication (yes/no), by replacing current depression with lifetime depression, or by removing use of antidepressant medication (ESM Tables 6–8). Only when physical activity was added to model 3 did the associations change slightly (ESM Table 9). However, information on physical activity was missing for 14% of all participants, which may explain these differences. Lastly, although not all associations reached statistical significance, scores on the individual tests were similar to those found on the domain score (ESM Table 10).

## Discussion

In the present study, we comprehensively investigated cross-sectional associations between plasma kynurenines and cognitive function in a large cohort of middle-aged individuals, in which the cohort was enriched with type 2 diabetes. In type 2 diabetes, higher levels of KYN, 3-HK, KA, XA and 3-HAA were associated with lower odds of cognitive impairment, and KA and XA were also associated with better executive function/attention. In prediabetes, only 3-HK and 3-HAA were associated with lower odds of cognitive impairment, and AA showed a non-linear association with odds of cognitive impairment and executive function/attention. No evidence of associations was found in participants with NGM.

### Glucose metabolism status, kynurenines and cognition

In general, the KYN pathway is more activated in an inflammatory environment, as exists in type 2 diabetes, resulting in upregulation of indoleamine 2,3-dioxygenase (IDO) [11] by cytokines (primarily IFN-γ). In line with this, previous studies found higher KYN, KA and XA concentrations in individuals with type 2 diabetes and higher levels of KYN, 3-HK and KA in those with diabetic retinopathy compared with individuals with NGM [10, 32]. This suggests a shift in kynurenine degradation towards KA and XA [10, 11]. Additionally, in a cross-sectional study in elderly participants, higher plasma levels of XA were associated with higher insulin resistance as well as higher odds of diabetes [33]. XA is believed to have diabetogenic effects through its ability to bind to insulin, forming chelate complexes that have a lower activity than insulin itself [11]. As a result, substantially increased levels of XA might contribute to insulin resistance and the development of type 2 diabetes.

Interestingly, our study showed that associations between higher levels of KYN, 3-HK, KA, XA, 3-HAA and lower odds for cognitive impairment were more pronounced in participants with type 2 diabetes, and associations with 3-HK and 3-HAA were also found in those with prediabetes. These findings have not been reported before and could suggest a protective role for these metabolites. This idea is in line with previous findings in Alzheimer’s disease [13, 14, 16, 17] suggesting that blood levels of KA, XA and 3-HAA are lower in affected individuals. Similarly, lower levels of 3-HAA have been reported in clinical samples with potential cognitive decline vs control samples, including end-stage Huntington’s disease, chronic brain injury and stroke [34] and higher levels of XA, being associated with a lower CVD mortality risk in a population study [35]. Clinical populations often show signs of low-grade chronic inflammation, yet our study suggests that the association between kynurenines and cognitive impairment is not explained by low-grade inflammation, as assessed by plasma levels of different cytokines.

We also observed a non-linear association for AA in prediabetes, suggesting that both high and low levels of AA are associated with higher odds of cognitive impairment. It is not clear what biochemical mechanisms are involved in this association, and it is generally assumed that the biological function of AA is inert [34, 36]. Obviously, more research into downstream kynurenines is needed.

### Potential underlying mechanisms

KA is an endogenous antagonist of glutamate receptors and is known for its neuroprotective effects because of its anti-glutamatergic and anti-inflammatory properties [12]. Glutamate is the primary excitatory neurotransmitter in the brain, and at normal physiological levels serves a vital role in neuronal plasticity and cognitive function. In contrast, extracellular glutamate overactivity can lead to excitotoxicity in Alzheimer’s disease and other neurodegenerative diseases, and the *N*-methyl-d-aspartate (NMDA) receptor antagonist memantine is a first line treatment for moderate to severe Alzheimer’s disease [37]. XA has a chemical structure similar to KA and can lead to a net reduction in extracellular glutamate levels [38], which could explain the positive associations of KA and XA with cognitive function in our study. 3-HAA also has anti-inflammatory properties by modulation of cytokine release and showed protective effects in pre-clinical disease models [39]. Moreover, KA, XA, 3-HAA and 3-HK are all antioxidants [36, 38] and can protect against oxidative stress, one of the important mechanisms involved in the pathogenesis of both type 2 diabetes and Alzheimer’s disease [8, 40]. At the same time, KYN, 3-HK and 3-HAA all possess some excitotoxic and neurodegenerative properties, including the induction of apoptosis of cells serving important roles in immune response [39]. The effects of these metabolites are thus likely pleiotropic and as such remain controversial [36], although our current data suggest that the neuroprotective effects predominate in prediabetes and type 2 diabetes.

### No evidence of associations in participants with NGM

We found no evidence of associations between kynurenines and cognitive function in the group with NGM, which is in line with previous cross-sectional findings in older adults from the Hordaland Health Study [19]. Both the Hordaland Health Study and the Maastricht Study are population-based studies in which the variation in concentrations of kynurenines is assumed to reflect that of the normal variation. In apparently healthy participants (i.e. participants without prediabetes or type 2 diabetes), this variation might be too subtle to lead to marked differences in cognitive ability. A more prolonged dysregulation of kynurenine metabolic pathways, as seen in type 2 diabetes and in the prediabetic stage, might be needed to result in neurogenerative and cerebrovascular pathology and cognitive decline. Contrary to the Hordaland Health Study, we did not find evidence for associations of KTR or neopterin with cognitive function. This might be explained by the younger average age in the Maastricht Study, with participants being age 40–75 years compared with 70–72 years in the Hordaland Health Study. Concentrations of kynurenines generally increase with age [14, 16], so one might expect stronger associations between them in older individuals. However, age did not modify the associations between concentrations of kynurenines and cognitive function in our study.

### Strengths and limitations

Strengths of the present study include a large sample size with a deep phenotyping approach, which allowed us to investigate the association between components of the kynurenine pathway and cognitive function while adjusting for a broad range of potential confounders. Additionally, in the Maastricht Study a large panel of fasting plasma kynurenines has been determined, and the extensive cognitive assessment allowed for investigation of a broad range of cognitive functions. The enrichment of the study population with type 2 diabetes allowed for well-powered interaction analyses. However, as analyses were exploratory and were not corrected for multiple testing, results should be interpreted with caution and be repeated in large prospective studies. Additionally, the use of a psychometric definition to determine cognitive impairment could be considered a limitation. However, this definition was based on domain scores incorporating multiple tests commonly used in epidemiological studies that are sensitive enough to pick up variations in cognitive functions. Furthermore, regression-based norm scores were used, taking into account age, sex and education level. Lastly, this study measured concentrations of kynurenines in plasma samples, and might not accurately reflect concentrations in the brain. However, recent studies suggest that peripheral concentrations of several kynurenine metabolites are positively correlated with their corresponding levels in cerebrospinal fluid in time-linked samples taken from individuals with Alzheimer’s disease and healthy control individuals [16, 17].

### Conclusion

Altogether, these cross-sectional results suggest that higher plasma concentrations of several kynurenines were associated with lower odds of cognitive impairment and higher levels of domain-specific cognitive functions, particularly in participants with type 2 diabetes. Future prospective studies should verify current results by investigating plasma and cerebrospinal fluid concentrations and more direct brain markers of Alzheimer’s disease, and by investigating larger groups of individuals with different levels of cognitive impairment.

## Supplementary information

ESM (PDF 225 kb)

## Data Availability

The dataset used in the present study was derived from the Maastricht Study. Upon reasonable request and with permission of the Maastricht Study management team, this dataset is available from the corresponding author.

## Abbreviations

AA: Anthranilic acid
CRP: C-reactive protein
3-HAA: 3-Hydroxyanthranilic acid
3-HK: 3-Hydroxykynurenine
IDO: Indoleamine 2,3-dioxygenase
KA: Kynurenic acid
KTR: Kynurenine/tryptophan ratio
KYN: Kynurenine
NGM: Normal glucose metabolism
PLP: Pyridoxal 5′-phosphate
QA: Quinolinic acid
SAA: Serum amyloid A
sICAM: Soluble intercellular adhesion molecule-1
TDO: Tryptophan 2,3-dioxygenase
TRP: Tryptophan
XA: Xanthurenic acid
