# Supplementary material for: Associations between plasma kynurenines and cognitive function in individuals with normal glucose metabolism, prediabetes and type 2 diabetes: the Maastricht Study
Source: Diabetologia. 2021 Aug 19;64(11):2445–57. doi: 10.1007/s00125-021-05521-4 (PMC8494700; doi:10.1007/s00125-021-05521-4)
Supplement: Supplementary file 1 — (PDF 225 kb) [file 125_2021_5521_MOESM1_ESM.pdf]

Table of contents

Electronic Supplementary material (ESM) Tables

ESM Table 1 General characteristics, cognitive domain scores and kynurenine concentrations of study participants by glucose metabolism status.

ESM Table 2 Concentrations of kynurenines, inflammation markers and B-vitamins of study participants by glucose metabolism status.

ESM Table 3 Interaction analyses of kynurenine concentrations with glucose tolerance status, age or sex in main model 2.

ESM Table 4 Association between kynurenic acid, xanthurenic acid and cognitive domain scores in participants with type 2 diabetes, after controlling for covariates.

ESM Table 5 Association between tryptophan, kynurenines and ratios and cognitive impairment, after controlling for additional covariates.

ESM Table 6 Association between tryptophan, kynurenines and ratios and cognitive impairment, after replacing hypertension with systolic blood pressure and hypertension medication.

ESM Table 7 Association between tryptophan, kynurenines and ratios and cognitive impairment, after replacing current depression with lifetime depression.

ESM Table 8 Association between tryptophan, kynurenines and ratios and cognitive impairment, after removing anti-depressive medication.

ESM Table 9 Association between tryptophan, kynurenines and ratios and cognitive impairment, additionally adjusted for physical activity.

ESM Table 10 Association between kynurenic acid and xanthurenic acid and cognitive test scores, in participants with type 2 diabetes.

ESM Figures

ESM Figure 1 Flowchart of Maastricht study

ESM Table 1 General characteristics, cognitive domain scores and kynurenine concentrations of study participants by glucose metabolism status

Characteristics	NGM (n = 957)	Prediabetes (n = 495)	Type 2 diabetes (n = 905)	p value
Demographics				
Age, years	58.1 ± 7.9	61.6 ± 7.6	62.6 ± 7.6	<0.001
Men, n *(%)*	404 (42.2)	268 (54.1)	612 (67.6)	<0.001
Educational level, n *(%)*				<0.001
Low	258 (27.0)	172 (34.7)	403 (44.5)	
Intermediate	272 (28.4)	142 (28.7)	246 (27.2)	
High	416 (43.5)	168 (33.9)	226 (25.0)	
Lifestyle factors				
Smoking, n *(%)*				<0.001
Never	379 (39.6)	145 (29.3)	244 (27.0)	
Former	471 (49.2)	282 (57.0)	488 (53.9)	
Current	99 (10.3)	61 (12.3)	143 (15.8)	
Alcohol use, n *(%)*				<0.001
None	133 (13.9)	78 (15.8)	255 (28.2)	
Low	545 (56.9)	259 (52.3)	449 (49.6)	
High	268 (28.0)	150 (30.3)	171 (18.9)	
Physical activity (h/week)	14.9 ± 8.1	14.0 ± 7.9	12.2 ± 7.7	<0.001
Cardiovascular risk factors				
BMI, kg/m^2	25.5 ± 3.7	27.8 ± 4.3	29.8 ± 5.0	<0.001
Systolic BP, mmHg	130.6 ± 17.2	137.6 ± 17.0	142.4 ± 17.9	<0.001
Diastolic BP, mmHg	74.9 ± 9.8	78.2 ± 9.6	77.2 ± 9.5	<0.001
eGFR, ml min^{-1} 1.73m^{-2}	90.2 ± 13.2	86.9 ± 13.9	85.0 ± 17.3	<0.001
Total/ HDL cholesterol ratio	5.61 ± 1.03	5.44 ± 1.15	3.74 ± 1.16	<0.001
Diseases, n *(%)*				
Hypertension	384 (40.1)	313 (63.2)	751 (83.0)	<0.001
History of CVD	109 (11.4)	66 (13.3)	239 (26.4)	<0.001
Depression	33 (3.4)	13 (2.6)	56 (6.2)	0.002
Medication use, n *(%)*				
Lipid-modifying agent	154 (16.1)	173 (34.9)	673 (74.4)	<0.001
Antihypertensive agent	203 (21.2)	220 (44.4)	653 (72.2)	<0.001

Antidepressant	55 (5.7)	44 (8.9)	74 (8.2)	0.044
Cognitive impairment	147 (15.4)	78 (15.8)	214 (23.6)	<0.001
Cognitive domain score				
Memory	0.17 ± 0.95	−0.03 ± 0.94	−0.35 ± 0.93	<0.001
Information processing	0.19 ± 0.74	−0.02 ± 0.76	−0.32 ± 0.79	<0.001
Executive function/ Attention	0.13 ± 0.74	−0.04 ± 0.77	−0.29 ± 0.84	<0.001

Data are presented as n *(%)* or mean ± SD

One-way ANOVA and χ^2 tests were used to investigate differences in characteristics between participants with normal glucose metabolism, prediabetes and type 2 diabetes. One of the participants had a diagnosis of type 1 diabetes and was excluded

ESM Table 2 Concentrations of kynurenines, inflammation markers and B-vitamins of study participants by glucose metabolism status

Characteristics	NGM (n = 957)	Prediabetes (n = 495)	Type 2 diabetes (n = 905)	p value
Metabolite levels				
TRP, μmol/L	63.20 (57.50–69.00)	63.40 (57.40–70.00)	61.70 (54.90–69.75)	0.034
KYN, μmol/L	1.57 (1.36–1.81)	1.67 (1.45–1.98)	1.69 (1.45–1.99)	<0.001
3-HK, nmol/L	39.20 (32.90–47.00)	42.80 (35.20–50.38)	44.20 (36.20–54.33)	<0.001
KA, nmol/L	48.00 (38.90–59.15)	55.10 (44.10–68.00)	56.20 (44.25–72.70)	<0.001
XA, nmol/L	13.40 (10.05–17.60)	15.30 (11.50–19.60)	14.70 (10.45–19.15)	<0.001
AA, nmol/L	14.20 (11.80–17.10)	14.80 (12.70–17.60)	15.55 (12.60–18.70)	<0.001
3-HAA, nmol/L	33.60 (27.40–41.20)	39.75 (31.33–48.30)	41.80 (32.98–52.23)	<0.001
QA, nmol/L	363.0 (302.0–443.0)	411.0 (326.0–514.0)	415.0 (328.0–535.0)	<0.001
Ratios				
KTR	24.82 (21.75–28.85)	26.34 (22.55–31.10)	26.96 (22.67–32.47)	<0.001
KA/QA	0.132 (0.106–0.164)	0.132 (0.105–0.170)	0.134 (0.109–0.170)	0.336
Low-grade inflammation[a]	-0.22 ± 0.56	0.11 ± 0.61	0.31 ± 0.66	<0.001
Inflammation markers				
Neopterin, nmol/L	15.61 (13.14–18.92)	16.60 (13.71–19.90)	17.78 (14.80–21.77)	<0.001
CRP, μg/ml	1.02 (0.49–2.21)	1.68 (0.80–3.55)	1.65 (0.75–3.76)	<0.001
SAA, μg/ml	2.96 (1.81–4.94)	3.64 (2.30–5.78)	3.59 (2.27–6.27)	<0.001
sICAM-1, ng/ml	319.5 (280.0–374.8)	343.4 (298.2–405.2)	364.5 (313.9–430.3)	<0.001
IL-6, pg/ml	0.49 (0.33–0.75)	0.65 (0.45–0.96)	0.79 (0.55–1.16)	0.004
IL-8, pg/ml	3.81 (3.10–4.68)	4.21 (3.34–5.35)	5.01 (4.04–6.38)	<0.001

TNF-α, pg/ml	2.08 (1.82–2.40)	2.23 (1.92–2.59)	2.43 (2.09–2.86)	<0.001
B-vitamins, nmol/L				
PLP	64.00 (44.70–93.45)	59.40 (42.60–86.00)	51.90 (36.80–73.45)	<0.001
Riboflavin	13.60 (8.89–21.70)	14.00 (9.09–22.10)	13.30 (8.37–22.75)	0.266

Data are presented as median (IQR) or mean ± SD

One-way analysis of variance (ANOVA) was used to investigate differences in characteristics between participants with normal glucose metabolism, prediabetes and type 2 diabetes. One of the participants had a diagnosis of type 1 diabetes and was excluded

[a]Composite score of CRP, SAA, sICAM-1, IL-6, IL-8 and TNF-α (transferred into z-scores and averaged).

ESM Table 3 Interaction analyses of kynurenine concentrations with glucose tolerance status, age or sex in main model 2

	Cognitive impairment		
	Glucose tolerance status	Age	Sex
TRP	0.304	0.701	0.605
KYN	0.598	0.913	0.911
3-HK	0.059	0.091	0.324
KA	0.630	0.591	0.794
XA	0.027*	0.918	0.328
AA	0.048*	0.198	0.966
3-HAA	0.030*	0.898	0.988
QA	0.746	0.429	0.778
KTR	0.903	0.806	0.711
KA/QA	0.267	0.307	0.517
Neopterin	0.437	0.320	0.889

Data are presented as p values

All analyses were adjusted for age, sex, educational level, eGFR, glucose tolerance status, BMI, total cholesterol/HDL-cholesterol ratio, lipid-modifying medication use, alcohol consumption and smoking behaviour (model 2)

*p<0.05

ESM Table 4 Association between kynurenic acid, xanthurenic acid and cognitive domain scores in participants with type 2 diabetes, after controlling for covariates

	Domain scores		
	Memory	Processing speed	Executive functioning and attention
Metabolites			
KA			
Model 1[a]	0.05 (-0.01, 0.11)	0.06 (0.01, 0.11)*	0.08 (0.03, 0.14)**
Model 2[b]	0.03 (-0.03, 0.09)	0.05 (-0.00, 0.10)	0.07 (0.02, 0.13)*
Model 3[c]	0.03 (-0.03, 0.09)	0.04 (-0.01, 0.09)	0.06 (0.01, 0.12)*
XA			
Model 1[a]	0.04 (-0.02, 0.09)	0.03 (-0.01, 0.08)	0.08 (0.03, 0.13)**
Model 2[b]	0.02 (-0.04, 0.07)	0.02 (-0.03, 0.06)	0.06 (0.01, 0.11)*
Model 3[c]	0.01 (-0.04, 0.07)	0.01 (-0.03, 0.06)	0.05 (0.00, 0.10)*

Data are presented as β per SD (95% CI)

[a]Model 1: adjusted for age, sex, educational level and eGFR

[b]Model 2: model 1 + BMI, total cholesterol/HDL-cholesterol ratio, lipid-modifying medication use, alcohol consumption and smoking behaviour

[c]Model 3: model 2 + current depressive episode, anti-depressant use, history of CVD, hypertension, B vitamins (PLP, riboflavin) and low-grade inflammation (composite score of CRP, SAA, sICAM-1, IL-6, IL-8 and TNF-α)

*$p<0.05$, **$p<0.01$

ESM Table 5 Association between tryptophan, kynurenines and ratios and cognitive impairment, after controlling for additional covariates

	Cognitive impairment		
	NGM	Prediabetes	Type 2 diabetes
TRP	1.04 (0.84, 1.28)	0.80 (0.60, 1.08)	0.91 (0.77, 1.07)
KYN	1.01 (0.78, 1.32)	0.88 (0.63, 1.22)	0.80 (0.66, 0.98)*
3-HK	1.24 (0.92, 1.67)	0.51 (0.30, 0.84)**	0.84 (0.69, 1.02)
KA	0.94 (0.70, 1.26)	1.19 (0.84, 1.69)	0.82 (0.69, 0.99)*
XA	1.04 (0.84, 1.29)	0.70 (0.50, 0.97)*	0.75 (0.63, 0.90)**
AA	0.77 (0.57, 1.05)	[a]	0.89 (0.73, 1.09)
3-HAA	1.01 (0.76, 1.33)	0.58 (0.39, 0.85)**	0.73 (0.60, 0.89)**
QA	0.91 (0.69, 1.18)	0.63 (0.40, 1.01)	0.82 (0.65, 1.04)
KTR	1.00 (0.75, 1.35)	1.01 (0.73, 1.41)	0.89 (0.73, 1.08)
KA/QA	1.00 (0.80, 1.25)	1.34 (1.04, 1.74)*	0.94 (0.79, 1.11)
Neopterin	0.98 (0.73, 1.31)	1.18 (0.83, 1.66)	1.05 (0.87, 1.26)

Data are presented as OR per SD (95% CI)

Model 3: model 2 + current depressive episode, anti-depressant use, history of CVD, hypertension, B vitamins (PLP, riboflavin) and low-grade inflammation (composite score of CRP, SAA, sICAM-1, IL-6, IL-8 and TNF-α) (*n*=2221)

[a]Association was non-linear according to likelihood ratio test and visual inspection

*p<0.05, **p<0.01

ESM Table 6 Association between tryptophan, kynurenines and ratios and cognitive impairment, after replacing hypertension with systolic blood pressure and hypertension medication

	Cognitive impairment		
	NGM	Prediabetes	Type 2 diabetes
TRP	1.03 (0.83, 1.28)	0.81 (0.61, 1.09)	0.91 (0.77, 1.07)
KYN	1.00 (0.77, 1.30)	0.87 (0.63, 1.22)	0.80 (0.65, 0.98)*
3-HK	1.23 (0.92, 1.66)	0.51 (0.31, 0.85)*	0.83 (0.68, 1.02)
KA	0.94 (0.70, 1.26)	1.21 (0.86, 1.72)	0.81 (0.68, 0.98)*
XA	1.04 (0.83, 1.29)	0.71 (0.51, 0.99)*	0.75 (0.62, 0.90)**
AA	0.77 (0.56, 1.05)	[a]	0.90 (0.74, 1.10)
3-HAA	1.00 (0.76, 1.32)	0.60 (0.41, 0.88)*	0.73 (0.60, 0.89)**
QA	0.90 (0.69, 1.18)	0.64 (0.40, 1.02)	0.82 (0.65, 1.04)
KTR	1.00 (0.74, 1.34)	1.00 (0.72, 1.38)	0.89 (0.73, 1.08)
KA/QA	1.01 (0.81, 1.26)	1.37 (1.05, 1.78)*	0.93 (0.78, 1.10)
Neopterin	0.98 (0.73, 1.31)	1.15 (0.81, 1.61)	1.05 (0.87, 1.26)

Data are presented as OR per SD (95% CI)

Model 3: model 2 + current depressive episode, anti-depressant use, history of CVD, systolic blood pressure, anti-hypertensives, B vitamins (PLP, riboflavin) and low-grade inflammation (composite score of CRP, SAA, sICAM-1, IL-6, IL-8 and TNF-α)

[a]Association was non-linear according to likelihood ratio test and visual inspection

*p<0.05, **p<0.01

ESM Table 7 Association between tryptophan, kynurenines and ratios and cognitive impairment, after replacing current depression with lifetime depression

	Cognitive impairment		
	NGM	Prediabetes	Type 2 diabetes
TRP	1.01 (0.81, 1.26)	0.80 (0.60, 1.08)	0.91 (0.77, 1.08)
KYN	1.04 (0.80, 1.35)	0.85 (0.61, 1.19)	0.80 (0.65, 0.99)*
3-HK	1.26 (0.94, 1.71)	0.52 (0.31, 0.88)*	0.82 (0.67, 1.02)
KA	0.92 (0.68, 1.24)	1.17 (0.83, 1.66)	0.81 (0.67, 0.98)*
XA	1.05 (0.84, 1.31)	0.71 (0.50, 0.99)*	0.73 (0.60, 0.90)**
AA	0.75 (0.55, 1.03)	[a]	0.89 (0.72, 1.10)
3-HAA	1.00 (0.76, 1.33)	0.59 (0.39, 0.88)**	0.73 (0.60, 0.90)**
QA	0.92 (0.71, 1.18)	0.61 (0.38, 0.98)*	0.79 (0.62, 1.02)
KTR	1.06 (0.79, 1.43)	0.99 (0.71, 1.38)	0.87 (0.71, 1.08)
KA/QA	0.97 (0.77, 1.23)	1.34 (1.04, 1.74)*	0.93 (0.78, 1.12)
Neopterin	0.98 (0.73, 1.31)	1.18 (0.83, 1.67)	1.08 (0.89, 1.31)

Data are presented as OR per SD (95% CI)

Model 3: model 2 + lifetime depressive episode, anti-depressant use, history of CVD, hypertension, B vitamins (PLP, riboflavin) and low-grade inflammation (composite score of CRP, SAA, sICAM-1, IL-6, IL-8 and TNF-α)

[a]Association was non-linear according to likelihood ratio test and visual inspection

*p<0.05, **p<0.01

ESM Table 8 Association between tryptophan, kynurenines and ratios and cognitive impairment, after removing anti-depressant medication

	Cognitive impairment		
	NGM	Prediabetes	Type 2 diabetes
TRP	1.04 (0.84, 1.29)	0.80 (0.60, 1.07)	0.91 (0.77, 1.07)
KYN	1.01 (0.78, 1.31)	0.87 (0.63, 1.22)	0.80 (0.65, 0.98)*
3-HK	1.23 (0.92, 1.65)	0.51 (0.31, 0.85)**	0.83 (0.68, 1.02)
KA	0.94 (0.71, 1.26)	1.19 (0.84, 1.69)	0.82 (0.68, 0.98)*
XA	1.04 (0.84, 1.29)	0.70 (0.50, 0.97)*	0.75 (0.62, 0.90)**
AA	0.78 (0.57, 1.06)	[a]	0.89 (0.73, 1.09)
3-HAA	1.00 (0.76, 1.32)	0.58 (0.39, 0.86)**	0.73 (0.60, 0.89)**
QA	0.90 (0.69, 1.18)	0.63 (0.40, 1.01)	0.82 (0.65, 1.04)
KTR	1.00 (0.74, 1.34)	1.01 (0.73, 1.40)	0.89 (0.73, 1.08)
KA/QA	1.01 (0.81, 1.25)	1.34 (1.03, 1.73)*	0.94 (0.79, 1.11)
Neopterin	0.97 (0.73, 1.30)	1.18 (0.83, 1.66)	1.04 (0.87, 1.25)

Data are presented as OR per SD (95% CI)

Model 3: model 2 + current depressive episode, history of CVD, hypertension, B vitamins (PLP, riboflavin) and low-grade inflammation (composite score of CRP, SAA, sICAM-1, IL-6, IL-8 and TNF-α)

[a]Association was non-linear according to likelihood ratio test and visual inspection

**p*<0.05, ***p*<0.01

ESM Table 9 Association between tryptophan, kynurenines and ratios and cognitive impairment, additionally adjusted for physical activity

	Cognitive impairment		
	NGM	Prediabetes	Type 2 diabetes
TRP	1.02 (0.81, 1.29)	0.78 (0.56, 1.09)	0.90 (0.75, 1.08)
KYN	1.07 (0.81, 1.41)	0.90 (0.63, 1.28)	0.81 (0.65, 1.03)
3-HK	1.30 (0.95, 1.78)	0.55 (0.32, 0.94)*	0.86 (0.70, 1.05)
KA	1.07 (0.79, 1.45)	1.37 (0.93, 2.02)	0.88 (0.72, 1.07)
XA	1.10 (0.88, 1.39)	0.74 (0.51, 1.07)	0.79 (0.65, 0.97)*
AA	0.86 (0.63, 1.17)	[a]	0.93 (0.76, 1.14)
3-HAA	0.96 (0.71, 1.30)	0.64 (0.41, 0.98)*	0.80 (0.65, 0.99)*
QA	0.94 (0.73, 1.20)	0.56 (0.33, 0.96)*	0.85 (0.67, 1.09)
KTR	1.11 (0.81, 1.52)	1.04 (0.73, 1.48)	0.91 (0.74, 1.12)
KA/QA	1.07 (0.84, 1.36)	1.56 (1.17, 2.08)**	0.98 (0.80, 1.19)
Neopterin	1.03 (0.75, 1.40)	1.06 (0.73, 1.54)	1.10 (0.89, 1.36)

Data are presented as OR per SD (95% CI)

Model 3: model 2 + current depressive episode, anti-depressant use, history of CVD, hypertension, B vitamins (PLP, riboflavin), low-grade inflammation (composite score of CRP, SAA, sICAM-1, IL-6, IL-8 and TNF-α) and physical activity

[a]Association was non-linear according to likelihood ratio test and visual inspection

*p<0.05, **p<0.01

ESM Table 10 Association between kynurenic acid and xanthurenic acid and cognitive test scores, in participants with type 2 diabetes

	Cognitive test scores	
	CST C	Stroop interference
KA	-1.53 (-2.55, -0.51)**	-1.49 (-3.33, 0.36)
XA	-1.26 (-2.20, -0.32)**	-1.58 (-3.28, 0.13)

Data are presented as β per SD (95% CI)

Lower scores on CST C and Stroop interference indicate better cognitive performance

Model 2: adjusted for age, sex, educational level, eGFR, BMI, total cholesterol/HDL-cholesterol ratio, lipid-modifying medication use, alcohol consumption and smoking behaviour

**p<0.01

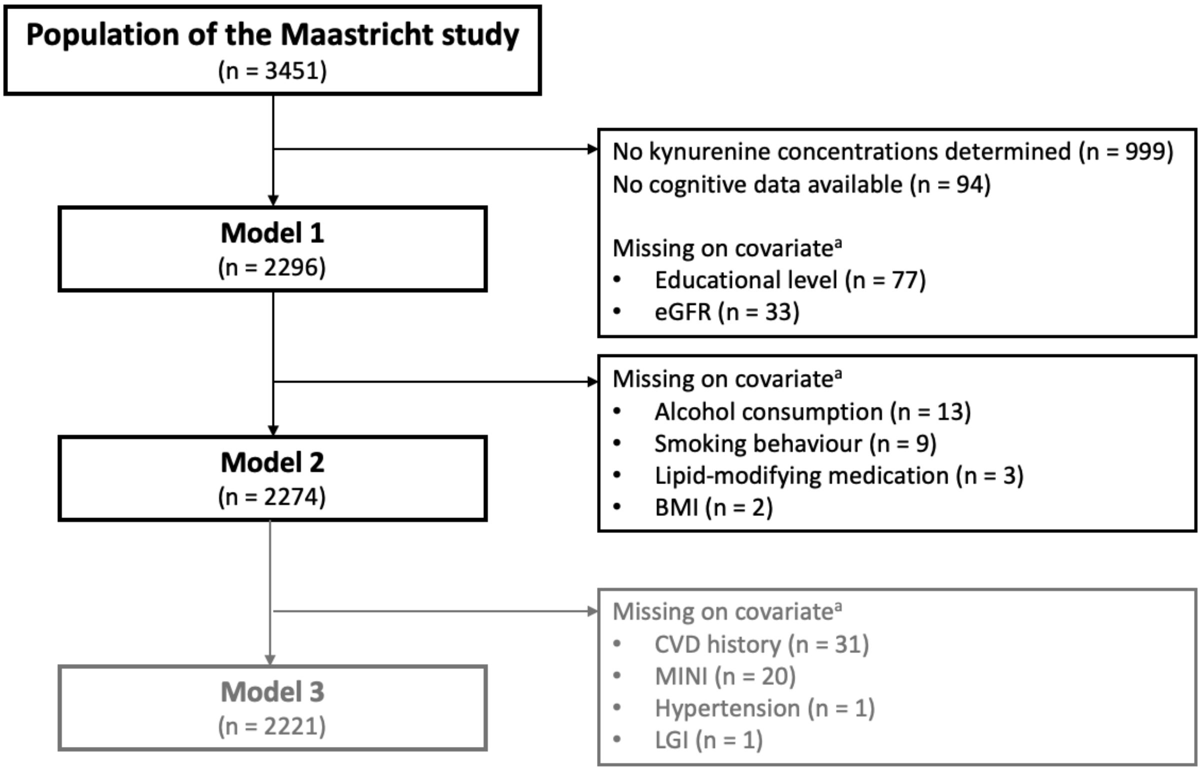

ESM Figure 1 Flowchart of Maastricht study

[a]Missing data on covariates are not mutually exclusive

MINI, Mini International Neuropsychiatric Interview; LGI, Low grade inflammation
